# Constructive remodeling of a synthetic endothelial extracellular matrix

**DOI:** 10.1038/srep18290

**Published:** 2015-12-21

**Authors:** Sewoon Han, Yoojin Shin, Hyo Eun Jeong, Jessie S. Jeon, Roger D. Kamm, Dongeun Huh, Lydia L. Sohn, Seok Chung

**Affiliations:** 1 The California Institute for Quantitative Biosciences, Stanley Hall, University of California, Berkeley, Berkeley, CA 94720, USA; 2 Department of Mechanical Engineering, Massachusetts Institute of Technology, 77 Massachusetts Ave., CambridgeMA 02139, USA; 3 School of Mechanical Engineering, Korea University, 145 Anam-ro, Seongbuk-gu, Seoul, 02841, South Korea; 4 Department of Mechanical Engineering, KAIST, 291 Daehak-ro, Yuseong-gu Daejeon 305-701, South Korea; 5 Department of Biological Engineering, Massachusetts Institute of Technology, 77 Massachusetts Ave., Cambridge MA 02139, USA; 6 Department of Bioengineering, University of Pennsylvania, 240 Skirkanich Hall, 210 South 33rd Street Philadelphia PA 19104, USA; 7 Department of Mechanical Engineering, Etcheverry Hall, University of California, Berkeley, Berkeley, CA 94720, USA

## Abstract

The construction of well-controllable *in vitro* models of physiological and pathological vascular endothelium remains a fundamental challenge in tissue engineering and drug development. Here, we present an approach for forming a synthetic endothelial extracellular matrix (ECM) that closely resembles that of the native structure by locally depositing basement membrane materials onto type 1 collagen nanofibers only in a region adjacent to the endothelial cell (EC) monolayer. Culturing the EC monolayer on this synthetic endothelial ECM remarkably enhanced its physiological properties, reducing its vascular permeability, and promoting a stabilized, quiescent phenotype. We demonstrated that the EC monolayer on the synthetic endothelial ECM neither creates non-physiological barriers to cell-cell or cell-ECM interactions, nor hinders molecular diffusion of growth factors and other molecules. The synthetic endothelial ECM and vascular endothelium on it may help us enter in a new phase of research in which various models of the biological barrier behavior can be tested experimentally.

The vascular endothelium, which lies at the blood-tissue interface, is both an important supplier of crucial metabolic factors and also a fundamental barrier, selectively limiting passage of blood plasma, circulating cells, and various pathogens^1^. The barrier function is mainly formed from cytoskeletons and cell-cell/matrix junctions^1^ and well known to be disrupted in tissues with diseases^2^,^3^. A number of *in vitro* assays employing an endothelial cell (EC) monolayer have been developed to assess its barrier function, including intravasation assay, extravasation assay, artificial membrane permeability assay, and Caco-2 permeability assay^4^,^5^. However, no straightforward method that recapitulates all features of the native vascular endothelium, including a lower, *in vivo*-like permeability value, cellular/extracellular components and a three-dimensional (3D) structure, is yet available^4^,^5^. In one such previously developed model, Cecchelli *et al.* (1999) reported that a brain capillary EC-astrocyte co-culture closely mimics the *in vivo* blood-brain barrier (BBB)^6^. In this two-dimensional (2D) model, bovine brain ECs were cultured on extracellular matrix (ECM)-coated porous membranes (luminal side) and astrocytes were cultured on the bottom surface (abluminal side) of a six-well plate, simultaneously. Other research groups have used similar methods to generate *in vitro* blood capillary models and other BBB models^7^,^8^. These previously described models have limitations, including a high vascular permeability value compared with that observed *in vivo*, diffusion instability of the Transwell devices used as a platform, and a non-physiological 2D structure (porous membrane or simple ECM-coated hard surface)^6^,^9^. 3D cell culture technology has recently come into the spotlight because of its *in vivo* relevance^10^; however, no previous study has shown *in vivo*-like vascular permeability values. In *in vivo*, EC monolayers are on the endothelial ECM, which consists of thin basement membrane (BM) on thick interstitial matrix and is necessary for stable maintenance of homeostasis in vascular endothelium^1^. Construction of *in vitro* models that genuinely reproduce the patho-physiological conditions of the *in vivo* vascular endothelium is increasingly important for creating various disease models, tissue engineering applications, and drug discovery efforts.

Here, we describe a synthetic endothelial ECM consisting of a thin local layer of basement membrane-coated type 1 collagen (BM-COL1) nanofibers, to mimic the thin basement membrane on the interstitial matrix *in vivo*. We present a simple method for generating 3D tube-like vascular endothelium surrounded by the BM-COL1 nanofibers. The layer with BM-COL1 nanofibers formulation incorporates the effects of ECM proteins and other stimuli and provides a means for recapitulating various phenotypes of the EC monolayer, including quiescent/tight EC monolayers under normal conditions and leaky/angiogenic capillary morphogenesis under specific pathological conditions.

Previously, we reported a method for incorporating COL1 hydrogel in a microfluidic device to mimic an interstitial matrix^11^,^12^,^13^. With this simple microfluidic approach, the COL1 can be gelled in a microfluidic channel by introduction of sodium hydroxide (NaOH) and thermal incubation. This COL1 gel is versatile and can be used as a fundamental ECM template in various 3D cell culture applications, including permeability assays and sprouting angiogenesis^13^, transendothelial migration^14^, cancer metastasis^15^, and other mono/co-culture models^16^,^17^. The distinctive feature of the EC monolayer that is most important for achieving reliable experimental results, particularly in permeability and transendothelial migration assays, is its barrier function. However, EC monolayers cultured on the COL1 are relatively leaky compared with the vasculature *in vivo*. We hypothesize that the low permeability^18^,^19^ might be due to the absence of an accompanying BM on the surface of COL1, as it is well known that the BM stabilizes the endothelium via orchestration of the actin cytoskeleton and recruitment of kinases (e.g., Src kinase and focal adhesion kinase [FAK]), a process that was triggered by integrin binding to the laminin (LN) that is the most abundant in BM^1^. We have created a synthetic endothelial ECM with a localized layer of BM-COL1 nanofibers through the introduction of diluted Growth Factor Reduced (GFR) Matrigel solution, which has similar components (e.g. LN and collagen type IV) to a natural BM (Fig. 1 and Supplementary Fig. S1,2).

A layer of BM-COL1 nanofibers, which contrasts sharply with normal COL1, is easily detected by phase-contrast microscopy with rabbit polyclonal anti-LN primary antibody and Alexa Fluor 488-conjugated goat anti-rabbit secondary antibody (Fig. 1b and Supplementary Fig. S2b). The GFR Matrigel starts to gel at temperatures above 10 °C. In order to prevent too fast gelation, which would lead to clogging within the microfluidic channel, we diluted the GFR Matrigel solution to 200 μg/mL with serum-free, microvascular endothelial cell-growth medium-2 (EGM2-MV) on an ice-cold plate. The BM-COL1 nanofiber layer thickness was measured by confocal microscopy (Fig. 1b), and found to be regulated by varying incubation times and concentration of the GFR Matrigel solution. At a fixed concentration of GFR Matrigel solution, the thickness is related to the incubation time alone,


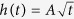


where *h(t)* is the resulting thickness of the BM-COL1 layer, *A* is a empirical constant that incorporates the fixed gelation temperature and concentration of the solution, and *t* is the incubation time (Fig. 1b). This relationship allowed us to control thickness of the layer of BM-COL1 nanofibers on a COL1 with a well-defined penetration depth (Fig. 1a,c). Furthermore, we found that the localized layer of BM-COL1 nanofibers generated stabilized human microvascular endothelial cell (hMVEC) monolayer (Fig. 1c).

To confirm that hMVECs stably form a 3D tube-like vascular endothelium in the presence of BM-COL1 nanofibers, we cultured hMVECs under two ECM conditions: synthetic endothelial ECM (localized layer of BM-COL1 nanofibers on normal COL1), and COL1-only. The seeding density in all experiments was fixed to 3 × 10^6^ cells/mL, and there was no significant difference in cell populations in culture day 5 (Supplementary Fig. S3). In the case of hMVECs grown on COL1-only, cells initially attached to the surface of the channel and the COL1 immediately after seeding, and gradually grew over a period of 5 days to form tube-like EC monolayer (Fig. 2a and Supplementary Fig. S4). However on culture day 5, the hMVECs detached from the surface (Fig. 2a). This detachment could be a result of a combination of mechanical contractions of the ECs, which might cause a leaky monolayer formation with defects in cell-cell junctions. However, hMVECs grown on the synthetic endothelial ECM formed stable 3D tube-like vascular endothelium without detaching and sprouting, even after 5 days of culture (Fig. 2b). To investigate integrity of junctional proteins in the 3D vascular endothelium grown on the synthetic endothelial ECM and COL1 only conditions, we evaluated expression of VE-cadherin, one of the major adherent junctions (AJs) (Fig. 2c and Supplementary Fig. S5)^1^. Confocal microscopic images showed that VE-cadherin was densely localized at every cell-to-cell contact of ECs on the synthetic endothelial ECM, whereas there is not a continuous network of VE-Cadherin at the cell-cell contact in COL1-only condition (Fig. 2c and Supplementary Movies S1-4). hMVECs persistently generated sprouts into the COL1 in the absence of the layer of BM-COL1 nanofibers (Fig. 2c, Supplementary Fig. S6, and Supplementary Movies S1,2). Eight sequential cross-sectional views and 3D confocal images show that the 3D tube-like vascular endothelium on the synthetic endothelial ECM was continuous, and appeared to be in a quiescent state (Fig. 2d–f). The layer of BM-COL1 nanofibers prominently suppressed behaviors that were phenotypically associated with the features observed in the COL1-only condition, such as detachment of hMVECs, frequent sprouting into the gel space, and sparsely distributed AJ proteins.

To quantify permeability of the vascular endothelium cultured on the synthetic endothelial ECM, we injected growth medium supplemented with fluorescein isothiocyanate (FITC)-dextran (MW: 40 kDa; 10μM) into the EC channels on culture days 4 and 5 (Fig. 3a,d,e). Two hours after addition of the FITC-dextran medium, we measured the fluorescence intensity profiles. In the vascular endothelium on the synthetic endothelial ECM, a sudden intensity drop was noticed, greater than that observed in the EC monolayer on COL1-only (Fig. 3a). The layer of BM-COL1 nanofibers thus did not impede the diffusion of FITC-dextran (Fig. 3b and Supplementary Fig. S7), possibly because the layer is thin and there is only a small difference between the diffusion coefficients of Matrigel (MAT) (5.76 × 10^−11^ m^2^/s) and COL1 (5.80 × 10^−11^ m^2^/s) (Supplementary Fig. S8)^20^,^21^,^22^. Also, the layer has large inter-fiber spaces filled with growth medium, and diffusion in the layer of BM-COL1 does not differ much from that in COL1. The synthetic endothelial ECM without an EC monolayer easily passed FITC-dextran molecules (Fig. 3c). The results lead us to conclude that the layer of BM-COL1 nanofibers does not disturb mass transfer, but does greatly enhance the tightness of the EC monolayer on it. The measured permeability value of the 3D tube-like vascular endothelium on the synthetic endothelial ECM (P = 8.305×10^−9^ m/s) was significantly lower than that in the COL1-only condition (P = 3.186×10^−7^m/s) (Fig. 3d)^23^. To the best of our knowledge, the acquired permeability is lower than all other values obtained by existing *in vitro* systems and are close to those measured *in vivo*^11^,^12^,^23^. We compared permeability values of EC monolayers cultured on the MAT, the synthetic endothelial ECM, and the COL1, and found that the permeability value of the MAT-only condition was much higher than that of the synthetic endothelial ECM condition (Fig. 3e). Instead of a diluted MAT solution, an LN solution was used to form LN-coated collagen (L-COL1) nanofibers in synthetic endothelial ECM and consequently gave similar low permeability value (P = 1.623×10^−8^m/s) as that of the layer of BM-COL1 nanofibers. It directly proved that LN was a main component in the BM-COL1 to generate tight vascular endothelium (Fig. 3f).

The layer of BM-COL1 nanofibers affected the tightness of hMVEC cell-cell junctions. We investigated mRNA levels of the major tight junction (TJ) proteins, claudin-1 (CLDN1), claudin-5 (CLDN5), and occludin (OCLN)^1^. Most blood capillaries contain TJs; and the TJs contribute to barrier function in specialized tissues, such as the brain and retina^24^,^25^. Quantitative real-time polymerase chain reaction (qRT-PCR) analyses revealed that CLDN1 and OCLN mRNA levels were substantially increased in the vascular endothelium on the synthetic endothelial ECM on culture day 3 (Fig. 3g). These results were also correlated with the protein level on culture day 5 through immunocytochemical staining (Fig. 3h–j). In both culture systems, the levels of CLDN5 mRNA were in similar range, but the fluorescence intensity of immunostained CLDN5 protein appeared slightly higher in the vascular endothelium on the synthetic endothelial ECM (Fig. 3g,i).

In addition to TJs between ECs, focal adhesions are also critical for maintaining strong EC-ECM adhesions, providing a mechanical connection to the endothelial ECM. Integrins, transmembrane receptors that interact with the cytoskeleton in the intracellular space through the linker proteins paxillin, vinculin, and talin, are the major structural components of focal adhesions^26^ and of which the extracellular domains bind to specific ECM proteins, including fibronectin, collagen, vitronectin, entactin, and LN^1^. Among these ECM proteins, we focused on LN, because it exhibits strong cell-adhesion activity, has the potential to stabilize the endothelium^27^, and is, as we have proved, a component in the BM-COL1 to generate tight vascular endothelium (Fig. 3f). Integrin α6 has a high affinity to LN^28^, and was found to show enhanced expression in the vascular endothelium on the synthetic endothelial ECM (Fig. 4a). LN-integrin-α6 interactions are known to lead to ‘outside-in signaling’ events that inform ECs of their environment and first encounters with surrounding molecules^1^, and further enhance LN-integrin α6 binding affinity and integrin clustering at the site of focal adhesions, which might subsequently strengthen adhesion to BM-COL1 nanofibers. Blocking antibody treatment of the GoH3 (against the integrin α6 β1^29^) significantly increased permeability of the vascular endothelium on the synthetic endothelial ECM (Fig. 4b). Taken together, we conclude that the LN-coated COL1 nanofibers in the synthetic endothelial ECM provides a beneficial effect, acting as an essential adhesion promoter and stabilizer of ECs.

A potential mechanism as to why the simple addition of the layer of BM-COL1 nanofibers between the COL1 interstitial matrix and ECs physiologically lowered the vascular permeability might be that the enhanced focal adhesion strength of ECs to BM-COL1 nanofibers promoted effective integrin clustering. These integrin clusters were subsequently stabilized by intracellular connections to the actin cytoskeleton, ultimately fostering the functional integrity of the endothelial barrier. Vascular permeability is known to be regulated by this cytoskeletal tension and retraction^1^. Diphosphorylation of regulatory myosin light chain 2 (RLC-pp) on Thr 18 and Ser 19 enhances the tension force, increasing the physical gap between EC-EC junctions and promoting hyperpermeability^30^. We found that over a 5-day culture period, the diphosphorylated form of MLC-2 was up-regulated near EC-EC contacts in the endothelial monolayer on the COL1-only (Fig. 4c,d). The layer of BM-COL1 nanofibers seem to suppress diphosphorylation of MLC-2. The tube-like vascular endothelium on the synthetic endothelial ECM has very low vascular permeability, but does not impede external stimulation or hinder transendothelial migration of neutrophils (Fig. 4e and Supplementary Fig. S9). ECs on the layer of BM-COL1 nanofibers respond to the 50 ng/mL of vascular endothelial growth factor (VEGF) gradient and form angiogenic sprouting and 3D capillary morphogenesis into the synthetic endothelial ECM (Fig. 4f–h and Supplementary Fig. S10).

In this work, we developed a simple, but reliable, method for coating LN on each COL1 nanofibers with densely localized the coated nanofibers to form a thin layer on the normal COL1 interstitial matrix. The method used natural ECM of COL1 as the basic structural nanofibers, and GFR Matrigel as a biomimetic BM source to produce the essential template for building stable, low-permeable and functional tube-like 3D vascular endothelium. From AJs to TJs and at the level of mRNA and basement membrane bound biologically active proteins, the vascular endothelium on the synthetic endothelial ECM showed physiological characteristics similar to those of the *in vivo*, exhibiting densely localized VE-cadherin junctions and highly expressed TJ proteins and corresponding mRNAs. The main advantage of this method is that the synthetic endothelial ECM is readily made by simply exposing 3D COL1 to the BM solution for no more than 1 hour. This is in contrast to other models using an EC monolayer, including *ex vivo* and *in vitro* 2D capillary models, which yield structures that hardly resemble *bona fide* blood capillaries. Moreover, many tests, such as measurements of vascular permeability and monitoring of transendothelial migration of immune cells or tumor cells and angiogenesis assays, can be performed in a same format with tube-like 3D vascular endothelium. Existing *ex vivo* and *in vitro* assays for investigating EC-drug and EC-ECM interactions and morphogenesis of vascular niches have failed to keep pace with research needs because of their structural vulnerabilities and physiological deficiencies, and increasing importance is being attached to engineered 3D blood capillaries. The method presented here, with its synthetic endothelial ECM based on natural materials, simple microfluidic technology, and physiological properties of the resulting 3D vascular endothelium, which are *in vivo*-like in all respects, provides an attractive alternative to the lagging technology of conventional models. We also anticipate that the method will help foster a new phase of research into various aspects of many epithelial barriers.

## Methods

### Microfluidic channel fabrication and preparation prior to filling with COL1 gel

The microfluidic channels were patterned by soft lithography. First, SU-8-100 photoresist (MicroChem, USA) was spin-coated onto a silicon wafer, which was then baked at 95 °C for 1 hour. The coated photoresist was selectively exposed to UV light through a mask bearing the microfluidic pattern, after which the exposed photoresist was developed in propylene glycol monomethyl ether acetate (PGMEA) photoresist developer (MicroChem, USA). Poly (dimethylsiloxane) (PDMS) solution containing Sylgard 184 silicone elastomer base and curing agent (weight ratio, 10:1; Dow Corning, USA) was cured on the patterned wafer at 80 °C for 1 hour in a dry oven. Inlet and outlet ports of all channels, including an EC channel, two side channels, and four ECM channels, were then opened with a biopsy punch. After autoclaving this patterned PDMS (upper part) and a glass coverslip (bottom part; Paul Marienfeld, Germany), the channel side of the upper part was irreversibly bonded to the bottom part by oxygen plasma treatment (CUTE; Femtoscience, South Korea). Next, a 1-mg/mL poly-D-lysine (PDL; MW: 30,000–70,000; Sigma-Aldrich, USA) solution in distilled deionized water (DDW) was immediately pipetted into the bonded device, after which the device was placed in a humidified 37 °C incubator for 4 hours. After washing away the excess PDL, the device was dried at 80 °C in an oven for more than 24 hours.

### Formation of COL1 in microfluidic channels

A mixed COL1 solution (BD Biosciences, USA), prepared by dilution in a mixture of 10X phosphate buffered saline (PBS; Thermo Scientific, USA) and DDW, was injected into ECM channels and allowed to gel in the incubator for 30 minutes. The pH of the COL1 solution was adjusted with a 0.5N NaOH solution to pH 10. Afterwards, all channels, excluding the ECM channels, were filled with EGM2-MV prior to the addition of BM solution into the EC channel.

### Formation of BM-COL1 layer on COL1

The BM solution is made of growth factor reduced (GFR) Matrigel (BD Biosciences, USA) and basal endothelial cell culture medium (EBM2, Lonza, USA). We diluted the GFR Matrigel to approximately 200 μg/mL with the EBM2. After gelling process of the COL1 in microfluidic channels, the BM solution was added to the EC channel and allowed to coat on the COL1 for 40 minutes. Excess the BM solution was then removed and washed thoroughly with EGM2-MV.

### Measurement of the thickness of the BM-COL1 layer

LNs were stained with rabbit polyclonal anti-laminin primary antibody (1:100; Abcam) and Alexa Fluor 488-conjugated goat anti-rabbit secondary antibody (1:200; Molecular Probes). The thickness of BM-COL1 layers in the synthetic endothelial ECM system were measured by a confocal microscopy (n = 4 ~ 6, per group).

### EC culture prior to seeding cells into the microfluidic channel

Cryopreserved hMVECs (third-passage), endothelial cell basal medium-2 (EBM-2), and medium supplements in the form of EGM-2-MV SingleQuots containing human epidermal growth factor (hEGF), hydrocortisone, gentamicin, VEGF, human basic fibroblast growth factor (hFGF-B), R^3^-insulin-like growth factor-1 (R^3^-IGF-1), ascorbic acid, heparin and fetal bovine serum (FBS), were obtained from Lonza (USA). hMVECs were expanded by growing in EGM-2MV (a mixture of EBM-2 and EGM2-MV SingleQuots) on tissue culture flasks for no more than five passages. These hMVECs were then cryopreserved at a concentration of 1 × 10^6^ cells/mL in EGM2-MV containing DMSO (10% v/v) and FBS (10% v/v) until use.

### EC culture in a microchannel

Before seeding hMVECs into the EC channel, a vial of cryopreserved hMVECs was thawed in a water bath. The cells were then seeded into a T75 tissue culture flask and incubated at 37 °C in a humidified CO_2_ incubator until reaching ~80% confluence. Growth medium was changed every 48 hours. HMVECs were detached by treating with a 0.05% trypsin/1 mM EDTA solution and prepared at a density of 2 × 10^6^ cells/mL. The center EC channel was filled with 50 μL of this cell suspension, and the device was placed in a 37 °C incubator for 30 minutes to allow cell attachment. Thereafter, non-adherent cells and cell debris were removed by placing 50 μL media droplets at all inlets and outlets of the microfluidic channels. The growth medium in all microfluidic channels was replaced daily with EGM2-MV.

### Numerical simulation of mass transfer

The concentration profiles of molecular diffusion from the EC channel to each side channel in devices containing COL1/EC monolayers or synthetic endothelial ECM/EC monolayers were illustrated by generating computational models using COMSOL Multiphysics 4.3 (COMSOL, Sweden) software. The diffusion coefficients for 40-kDa FITC-dextran in EGM2-MV, gelled COL1, gelled Matrigel, and the EC monolayer were assumed to be 8.00 × 10^−11^, 5.80 × 10^−11^, 5.76 × 10^−11^, and 1.57 × 10^−12^ m^2^/s, respectively (Supplementary Fig. S4). The transient diffusion profiles of FITC-dextran in the device from 0 to 12 hours were estimated by Fick’s second law, and the boundaries of the device were set to the no-mass-flow condition.

### Measurement of endothelial permeability

Absolute endothelial permeability was determined by adding 10 μM FITC-dextran (MW: 40 kDa) to the EC channel of the device 4 days after seeding ECs. Fluorescent images were acquired 2 hours after the addition of the FITC-dextran solution. Endothelial permeability was estimated from FITC-dextran diffusion, visualized by analyzing fluorescent images using ImageJ software. The concentration distribution of the FITC-dextran solution is governed by Fick’s first law, described by the following equation:


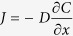


where ***J*** is flux, ***D*** is the diffusion coefficient, and ***x*** is the position.

Fick’s first law gives rise to the following formula:


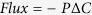


where ***P*** is permeability and ***C*** is concentration.

### Transmission electron microscopy (TEM) of COL1, synthetic endothelial ECM, and synthetic endothelial ECM with an EC monolayer

All materials used in this process were obtained from Sigma-Aldrich. The samples were fixed by incubating with 2.5% glutaraldehyde in 0.15 M 4-(2-hydroxyethyl)-1-piperazineethanesulfonic acid (HEPES) buffer at 4 °C for at least 24 hours. The samples were then postfixed in a 0.1 M sodium cacodylate trihydrate-buffered 1% osmium tetroxide (OsO_4_) solution for 1 hour and contrasted with 0.5 M Trizma maleate-buffered 0.5% uranylacetate. Afterwards, samples were dehydrated with graded series of ethanol solutions (25%, 50%, 75%, 95%, and 100%) in DDW. COL, synthetic endothelial ECM, and ECs in microfluidic channels were embedded with an Epoxy Embedding Medium Kit. Embedded samples were detached with the PDMS membrane, which had been treated with oxygen plasma for relatively short period, instead of the bottom part (glass coverslip) of the microfluidic devices. Ultrathin sections (60–70 nm) were prepared with an ultramicrotome (Ultracut UCT; Leica, Germany) and collected on 200-mesh grids. Sections were contrasted with uranylacetate and examined in a JEM 1010 transmission electron microscope (JEOL, Japan) operating at 60 kV. TEM images were recorded with a CCD camera (SC1000; Gatan, USA).

### qRT-PCR analyses

Total RNA was isolated from each sample (n = 4 per group) using the RNeasy Mini kit (Qiagen, USA). The RNA concentration was determined and normalized by measuring absorbance at 260 nm using a NanoDrop spectrophotometer (Thermo Scientific). RT reactions were performed using a High Capacity RNA-to-cDNA Kit (Invitrogen, USA). qRT-PCR measurements of gene expression were performed with Universal PCR Master Mix (Applied Biosystems, USA) using a StepOne Real-Time PCR System (Applied Biosystems). Gene expression in hMVECs in COL1 and synthetic endothelial ECM systems was quantified using TaqMan Gene Expression Assays (Applied Biosystems) for CLDN1 (Hs00221623_m1), CLDN5 (Hs00533949_s1), OCLN (Hs00170162_m1), and GAPDH (Hs02758991_g1). The mRNA levels of target genes were normalized to that of the endogenous reference (GAPDH), and relative differences in target gene expression were determined using the comparative Ct method. The normalized expression of each marker in hMVECs cultured in the COL1 and the synthetic endothelial ECM systems was expressed relative to that in the COL1 system on culture day 1.

### Immunocytochemistry

ECs cultured in microfluidic devices were fixed by adding 4% (w/v) paraformaldehyde (in PBS) into each reservoir of the devices and incubating for 15 minutes. Cells were permeabilized with 1% Triton-X100 (Sigma-Aldrich) in PBS for 10 minutes. After blocking with 20% Block Ace (in PBS) (Dainihon-Seiyaku, Japan) for 1 hour and washing the channels with PBS, solutions of rabbit polyclonal anti-laminin (1:100; Abcam, USA), rabbit polyclonal anti-CLDN1 (5 μg/mL; Abcam), rabbit polyclonal anti-CLDN5 (1:100; Abcam), rabbit polyclonal anti-OCLN (2 μg/mL; Abcam), and mouse monoclonal anti-integrin alpha 6 (1 μg/mL; Abcam) primary antibodies were introduced into the devices and incubated for 1 hour at room temperature. After washing with PBS, Alexa Fluor 488-conjugated goat anti-rabbit and goat anti-mouse IgG secondary antibodies (1:200; Molecular Probes, USA) were added to the device and incubated for 1 hour. Cell nuclei and actin filaments were counterstained with 4′, 6-diamidino-2-phenylindole (DAPI; Sigma-Aldrich) and rhodamine phalloidin (Sigma-Aldrich), respectively. Stained cells were observed under a fluorescence microscope (Axio Observer D1; Carl Zeiss, Germany) and a confocal microscope (LSM-700; Carl Zeiss).

### Neutrophil-like cell differentiation for an *in vitro* inflammation model

Human promyelocytic HL-60 cells (KCLB, South Korea) were grown in RPMI-1640 medium (Thermo Scientific) supplemented with 10% FBS (Lonza), 0.1 mM nonessential amino acids, 50 U/mL penicillin, and 50 μg/mL streptomycin at 37 °C in a humidified 5% CO_2_ incubator. The HL-60 cells were induced to undergo differentiation along the neutrophil lineage by seeding aliquots of HL-60 cell suspension (2 × 10^6^ cells/mL) onto a tissue culture flask and growing for 5 days in the presence of 1.25% dimethyl sulfoxide (DMSO). Complete differentiation of HL-60 cells into neutrophil-like cells (abbreviated dHL-60) was confirmed by nuclear segmentation and granule formation, spectrophotometry at 540 nm, and flow cytometry of FITC-conjugated monoclonal antibodies against CD11b, CD16, and CD33 (BD Biosciences). Thereafter, dHL-60 cells were cryopreserved at a concentration of 2 × 10^6^ cells/mL in FBS containing DMSO (10% v/v) until seeding into the EC channel.

### *In vitro* models

For the *in vitro* inflammation model, a cryovial of dHL-60 cells was quickly thawed in a 37 °C water bath. The dHL-60 cell suspension was prepared at a density of 1 × 10^6^ cells/mL, and 100 μL of this cell suspension was added to the confluent EC monolayer in the device at EC culture day 5. Then, 100 μL growth medium droplets containing N-formylmethionine leucyl-phenylalanine (fMLP; 10 nM), as a chemoattractant, were added to the side channels. For the *in vitro* angiogenesis model, growth medium supplemented with 50 ng/mL of VEGF and EGM-2MV (VEGF concentration: 20 ng/mL) were added to the confluent EC monolayer and the side channels of the device at EC culture day 5, and the media were changed every 24 hours for 3 days.

### Image processing

All images were processed and composites were made using the Adobe Design Premium CS6 software package. Where necessary, only contrast and brightness of the original images were minimally adjusted; all adjustments were within the linear range.

## Additional Information

**How to cite this article**: Han, S. *et al.* Constructive remodeling of a synthetic endothelial extracellular matrix. *Sci. Rep.* **5**, 18290; doi: 10.1038/srep18290 (2015).

## Supplementary Material

Supplementary Information

Supplementary Movie 1

Supplementary Movie 2

Supplementary Movie 3

Supplementary Movie 4

## Figures and Tables

**Figure 1:**
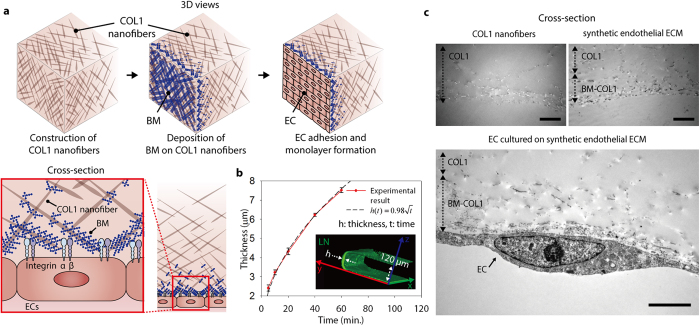
Generation of a 3D endothelial ECM. (**a**) Schematic overview of the deposition process. BM materials gradually attached onto the surface of the COL1 formed prior to the deposition process and remodel into a synthetic endothelial ECM. These illustrations were created from the area corresponding to the dotted rectangle in Supplementary Figure S.2b. (**b**) In the deposition process, layer thickness of BM-COL1 nanofibers (*h*) is controlled by incubation time (*t*) and follows a square-root relation (inset equation), at a fixed concentration of BM solution and incubation temperature. The confocal microscopic image (inset) was obtained from the area corresponding to the dotted rectangle in Supplementary Figure S.2b, which shows the formed layer of BM-COL1 nanofibers (green, LN). Error bars, ± SEM (*n* = 4 ~ 6). (**c**) Cross-sectional images of COL1 (upper left), synthetic endothelial ECM (upper right), and an EC on the surface of the synthetic endothelial ECM (lower image). Scale bars = 4 μm.

**Figure 2:**
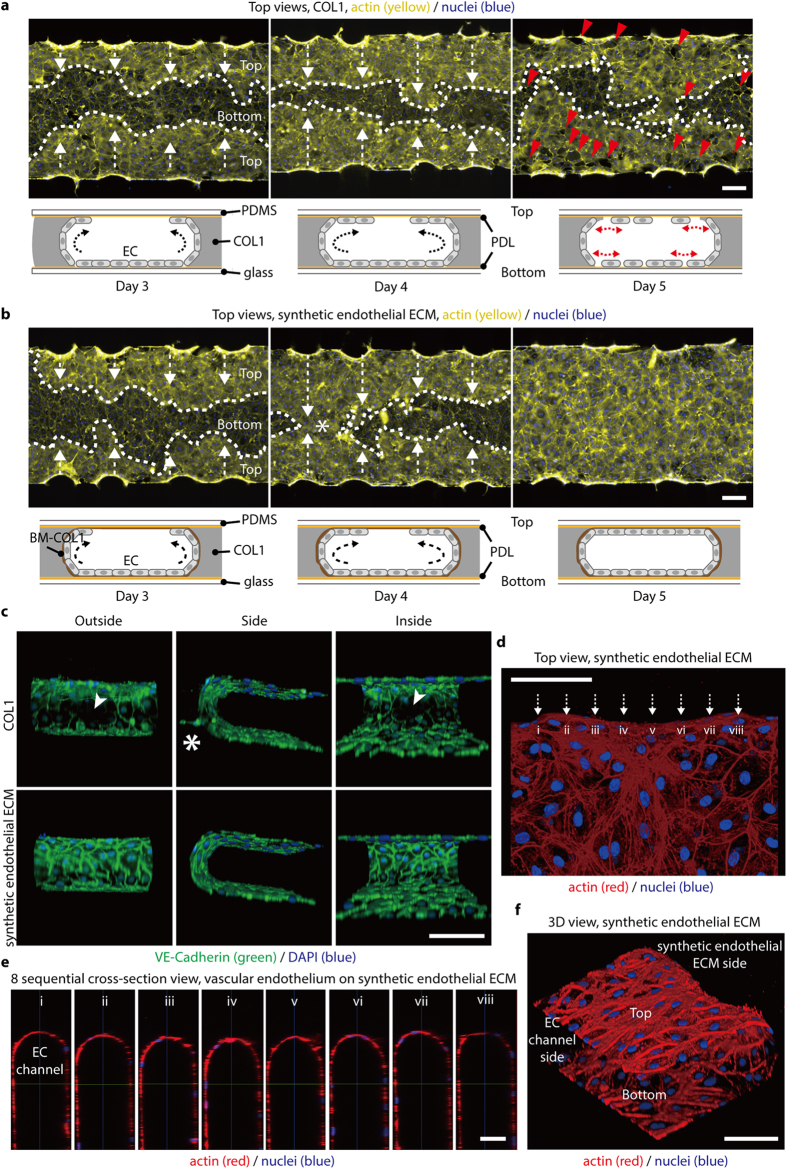
BM-COL1 greatly enhances the 3D characteristics of an *in vitro*-cultured, engineered blood capillary. (**a**,**b**) Fluorescence microscopic images and schematic depictions of blood capillary formation over time. (**a**) ECs (yellow, actin; blue, nuclei) cultured in the COL1 system easily detached from the top and bottom surfaces. Red arrowheads indicate the detached areas and white arrows indicate the growth direction of EC monolayers on the top surfaces. (**b**) ECs (yellow, actin; blue, nuclei) in the synthetic endothelial ECM system stably adhered to all areas. White arrows indicate the growth direction of EC monolayers on the top surfaces, and an asterisk indicates the merged area of both EC monolayer cultures from the top edge of each ECM channel. (**c**) VE-cadherin (green) AJs were densely localized at every EC-EC contact in the synthetic endothelial ECM system (lower images), but were deficient in the COL1 system (upper images). An asterisk in the upper panel indicates unintended sprouting angiogenesis, and a white arrowhead indicates the VE-cadherin deficiency in ECs cultured on the COL1. (**d**) Confocal microscopic images show that the engineered blood capillary (red, actin; blue, nuclei) cultured in the synthetic endothelial ECM system has no defects on any surface, including top, bottom, and BM-COL1 sides. (**e**) Eight sequential cross-sectional images taken from Fig. 2d (i-viii) show a perfect EC monolayer. (**f**) A 3D view of the engineered blood capillary shown in Fig. 2d,e. Scale bars, 200 μm (**a,b**), 100 μm (**c,d,f**), and 50 μm (**e**).

**Figure 3:**
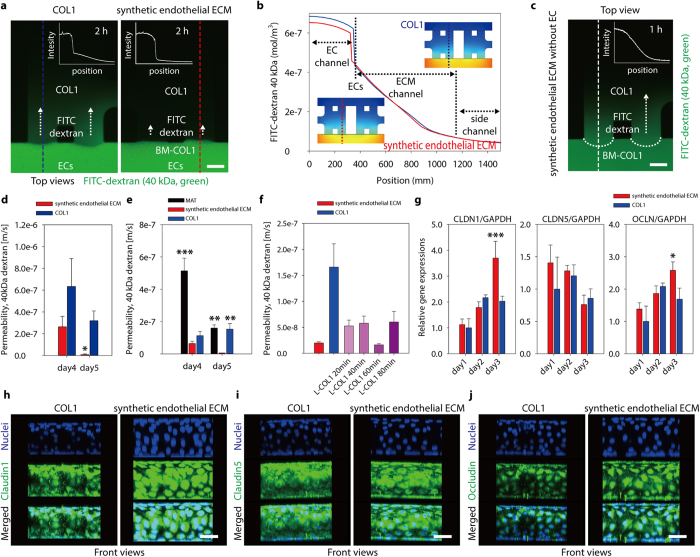
An *in vivo*-like barrier function in ECs cultured on an BM-COL1 layer was stably generated following the deposition of BM onto COL1 nanofibers. (**a**) Absolute permeability values of EC monolayers in the COL1 system (left) and the synthetic endothelial ECM system (right) were measured by introducing growth medium supplemented with 10 μM FITC-dextran (40 kDa, green) into the EC channel and monitoring FITC fluorescence. Inset graphs show intensity profiles of each dotted line (blue dotted line, COL1; red dotted line, synthetic endothelial ECM), and white arrows indicate the direction of diffusion. The images were taken 2 hours after the introduction of FITC-dextran-supplemented growth medium. Scale bar, 100 μm. (**b**) Simulated diffusion profiles at 2 hours after the introduction of FITC-dextran-supplemented growth medium in the COL1 (blue line) and the synthetic endothelial ECM (red line) systems containing an EC monolayer on each gel surface. (**c**) The fluorescence microscopic image shows the diffusion of FITC-dextran from an EC channel to a side channel passing through the BM-COL1 layer in the absence of an EC monolayer. The image was taken 1 hour after the addition of FITC-dextran-supplemented growth medium. The inset graph indicates the intensity profile of the white dotted line. Scale bar, 100 μm. (**d**) Absolute vascular permeability values for the synthetic endothelial ECM system (red bars) were dramatically reduced compared with those for the COL1 (*P = *0.029*) and approximated *in vivo* levels. Error bars, ± SEM (*n* = 4). (**e**) Absolute vascular permeability values for the MAT system were much higher than those for the synthetic endothelial ECM system (***P < *0.001* for the MAT on culture day4; **P = *0.002* for the MAT and **P = *0.005* for the COL1 on culture day5). Error bars, ± SEM (*n* = 8 ~ 24). (**f**) Absolute vascular permeability values of the synthetic endothelial ECM (BM-COL1), the COL1 (COL1), and the LN-coated COL1 (L-COL1) systems. For the generation of optimal layer for the L-COL1, we coated LNs onto the COL1 fibers for 20, 40, 60, and 80 min. in a humidified 37 °C incubator. Error bars, ± SEM (**P < 0.01 and ***P < 0.001, n = 5 ~ 18). (**g**) Levels of mRNA for the TJ proteins CLDN1, CLDN5, and OCLN in the COL1 (blue bars) and the synthetic endothelial ECM (red bars) systems, normalized to those of GAPDH, were acquired on culture days 1, 2, and 3 (*P = *0.016* and ***P < *0.001*). Error bars, ± SEM (*n* = 3 ~ 4). (**h–j**) Expression levels of CLDN1, CLDN5, and OCLN proteins in the COL1 and the synthetic endothelial ECM systems on culture day5 were determined by fluorescence microscopy (green, CLDN1/CLDN5/OCLN; blue, nuclei). Scale bars, 50 μm.

**Figure 4:**
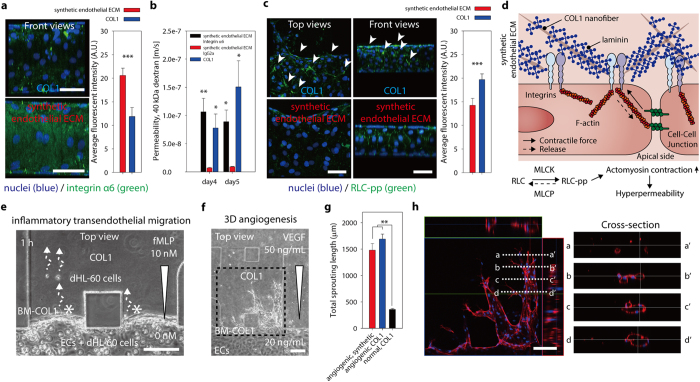
*In vivo*-like endothelial ECM-integrin cascade induced by BM-COL1 and applications of the synthetic endothelial ECM system. (**a**) Expression of integrin α6 (green) in COL1 and synthetic endothelial ECM systems. ECs in the synthetic endothelial ECM system strongly expressed integrin α6 on their cell membranes. Scale bar, 100 μm. (right) Average fluorescent intensities of the integrin alpha6 expressions in the synthetic endothelial ECM (red bar) and the COL1 (blue bar) systems at culture day5. Error bars represent standard deviation (***P = 0.001, n = 4 ~ 6). (**b**) Absolute vascular permeability values for the blocking antibody (the GoH3 rat monoclonal antibody against the integrin alpha 6 subunit) treated synthetic endothelial ECM system were significantly increased to the levels of the COL1 compared with those of negative control (IgG2a). (*P = 0.030, **P = 0.001 on culture day4; *P = 0.042 on culture day5). Error bars, ± SEM (n = 8 ~ 20). (**c**) Tension force was assessed by monitoring the expression levels of diphosphorylated regulatory myosin light chain 2 (RLC-pp; green). White arrows in the upper image indicate overexpressed RLC-pp in ECs cultured on COL1. (right) Average fluorescent intensities of the RLC-pp expressions in the synthetic endothelial ECM (red bar) and the COL1 (blue bar) systems at culture day5. Error bars represent standard deviation (***P = 0.001, n = 4 ~ 6). (**d**) Schematic of the mechanism underlying the stabilization and tightening of ECs by strengthening EC-ECM adhesion. (**e**) Neutrophil-like cells (differentiated human HL-60 promyelocytic leukemia cells [dHL-60]), could pass through the EC monolayer and even the BM-COL1 layer. The phase-contrast image was taken 1 hour after seeding dHL-60 cells. Scale bar, 100 μm. (**f**) The phase-contrast image shows sprouting angiogenesis in the presence of BM-COL1. The image was taken 3 days after stimulation with VEGF. (**g**). Total sprouting lengths of the sprouting angiogenesis in the synthetic endothelial ECM and the COL1 systems under the angiogenic condition (50-20 ng/ml of VEGF gradient) and the control condition (20-20 ng/ml, no VEGF gradient). Error bar represents standard error (**P = 0.006, n = 2). (**h**). A confocal microscopic image of the dotted square area in Fig. 4f. Scale bars, 100 μm (**a,c**) and 150 μm (**e,f,h**).
