# Supplementary material for: Constructive remodeling of a synthetic endothelial extracellular matrix
Source: Sci Rep. 2015 Dec 21;5:18290. doi: 10.1038/srep18290 (PMC4685304; doi:10.1038/srep18290)
Supplement: Supplementary Information [file srep18290-s1.doc]

**Supplementary Information**

**Article title:** Constructive remodeling of a synthetic endothelial extracellular matrix

**Authors:** Sewoon Han^1^, Yoojin Shin^2^, Hyo Eun Jeong^2^, Jessie S. Jeon^3^, Roger D. Kamm^4,5^,
Dongeun Huh^6^, Lydia L. Sohn^1,7^, and Seok Chung^2^*

**Affiliations of institutions:** ^1^The California Institute for Quantitative Biosciences, Stanley Hall, University of California, Berkeley, Berkeley, CA 94720, USA

^2^Department of Mechanical Engineering, Korea University, #512B, Innovation Hall, Anam, Seongbuk, Seoul, 136-713, South Korea,

^3^Departments of Mechanical Engineering, KAIST, 291 Daehak-ro, Yuseong-gu, Daejeon, 305-701, South Korea,

*^4^Departments of Mechanical Engineering and ^5^Biological Engineering, Massachusetts Institute of Technology, 77 Massachusetts Ave., NE47-321, Cambridge MA 02139, USA,*

^6^Department of Bioengineering, University of Pennsylvania, 240 Skirkanich Hall, 210 South 33^rd^ Street, Philadelphia, PA 19104, USA,

*^7^Department of Mechanical Engineering, Etcheverry Hall, University of California, Berkeley, Berkeley, CA 94720, USA*

**Corresponding author:** Professor Seok Chung

Address: #512B Innovation Hall, Korea University, Anam-dong, Sungbuk-gu, Seoul, South Korea

Email: [sidchung@korea.ac.kr](mailto:sidchung@korea.ac.kr)

Phone: +82-2-3290-3352

Fax: +82-3290-3352

**Supplementary Figure count: 10**

**Supplementary Movie count: 4**

**
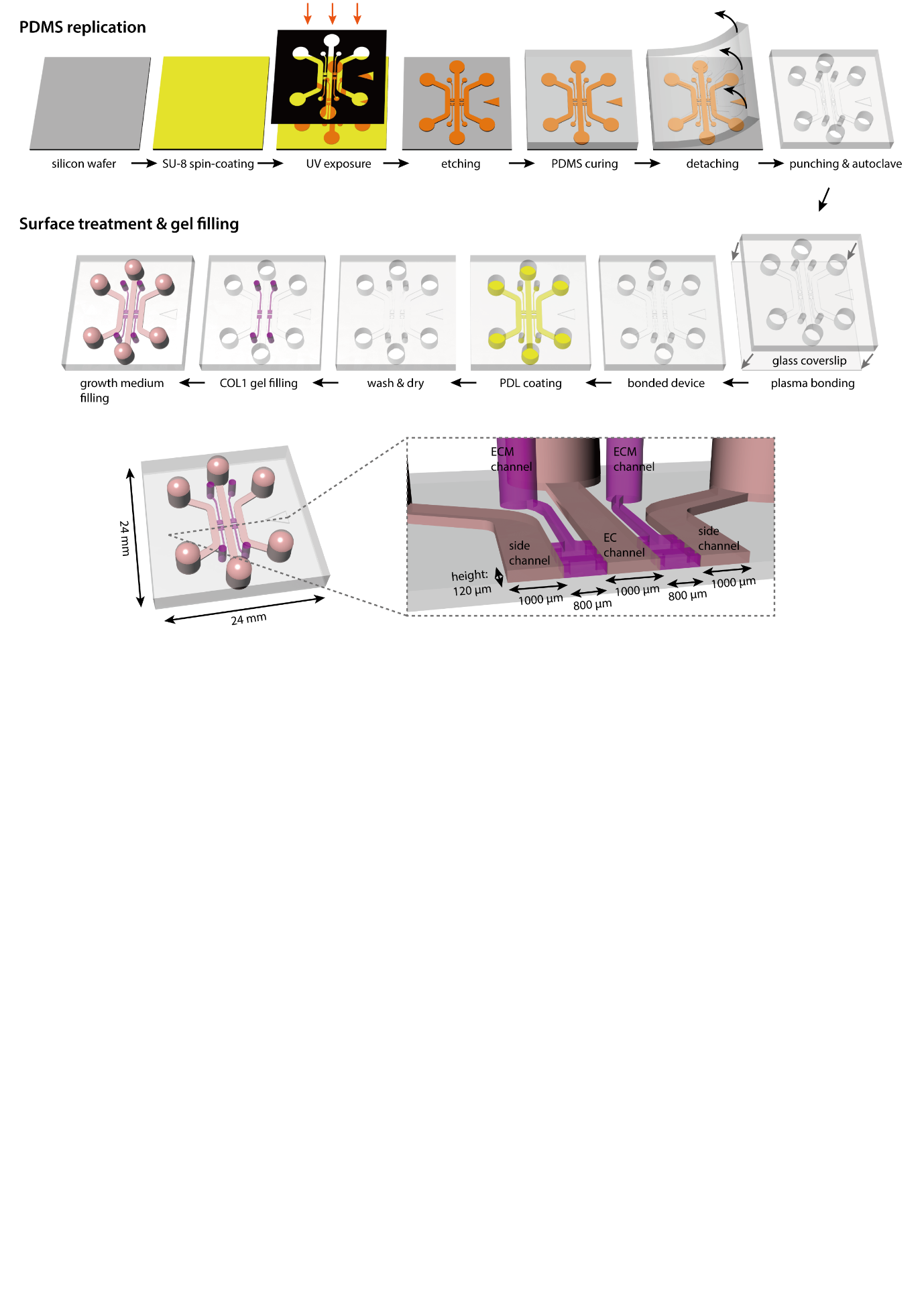
**

**Supplementary Figure 1|** Device preparation, including Poly (dimethylsiloxane) (PDMS) replication, surface treatment, and type 1 collagen (COL1) gel filling into the extracellular matrix (ECM) channels, prior to nano deposition procedure of basement membrane (BM) on COL1 nanofibers, termed BM-COL1. SU-8-100 photoresist (PR) was spin-coated onto a silicon wafer, which was then baked. The coated PR was selectively exposed to UV light through a mask bearing the microfluidic patterns. The exposed PR was developed in propylene glycol monomethyl ether acetate (PGMEA) photoresist developer. PDMS solution containing Sylgard 184 silicone elastomer base and curing agent was cured on the patterned. Inlet and outlet ports of all channels, including an endothelial cell (EC) channel, two side channels, and four extracellular matrix (ECM) channels, were then opened with a biopsy punch. After autoclaving the PDMS part and a glass coverslip, the PDMS part was irreversibly bonded to the bottom part by plasma bonding. Poly-D-lysine (PDL) solution was immediately pipetted into the bonded device, after which, the device was placed in a humidified 37 °C incubator. After washing away the excess PDL, the device was dried in an oven. A mixed COL1 solution, prepared by dilution in a mixture of 10X phosphate buffered saline (PBS) and DDW, was injected into ECM channels and gelated in the incubator. All channels, excluding the ECM channels, were filled with microvascular endothelial growth medium 2- (EGM2-MV) prior to the addition of basement membrane (BM) solution into the EC channel.

**
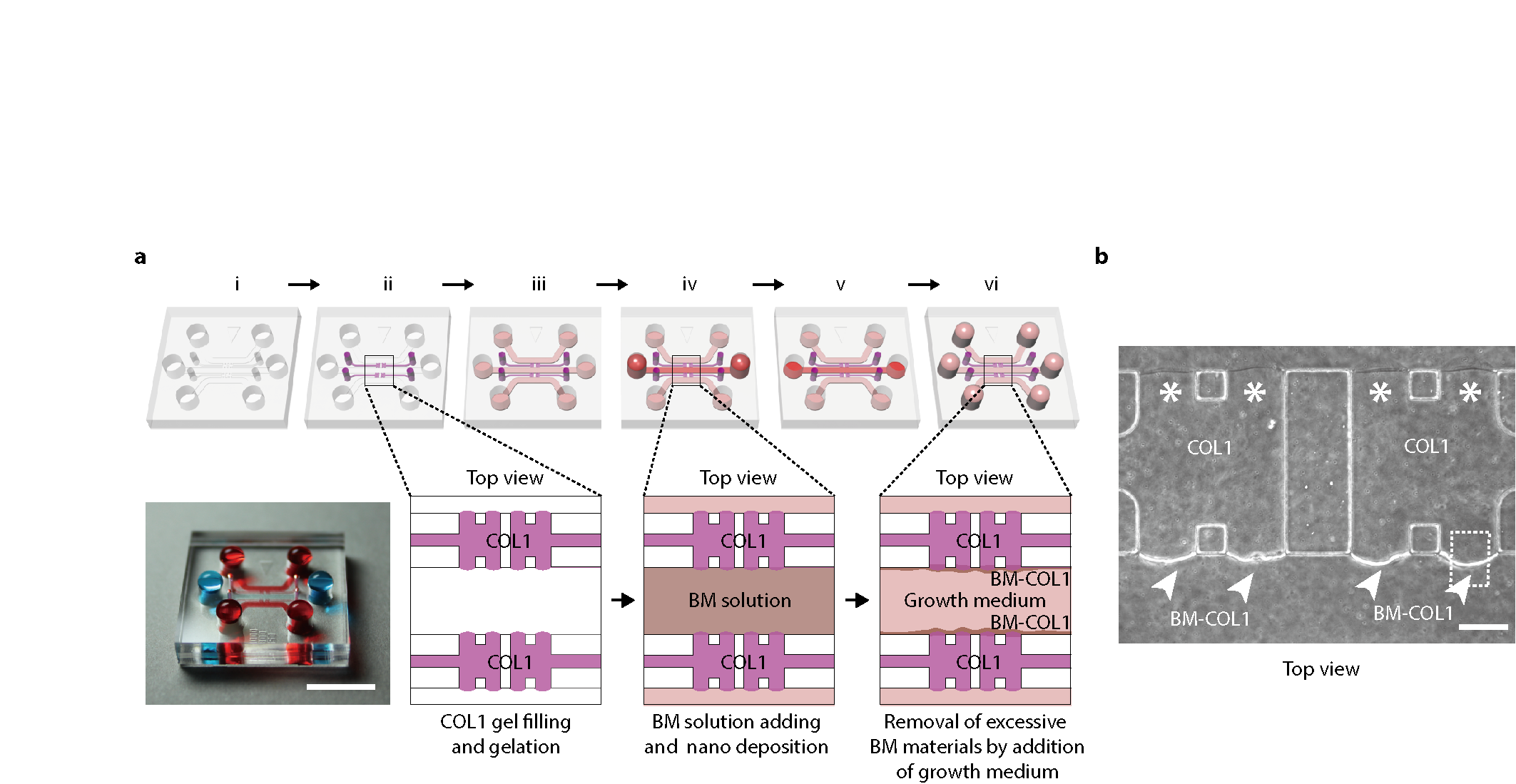
**

**Supplementary Figure 2| a**, Schematic of deposition of BM on COL1 nanofibers. (i) An autoclaved microfluidic device. (ii) The COL1 gel solution is introduced into ECM channels of the device and allowed to gel. (iii) An EC channel and both side channels are wetted with serum-free growth medium. (iv) The BM solution is added to the EC channel and allowed to coat. (v) The BM solution is removed after the deposition process. (vi) Excess BM material is removed by thoroughly washing with fresh serum-supplemented growth medium. Scale bar, 10 mm. **b**, Layer of BM-COL1 nanofibers (arrow heads) on the surface of the COL1 contrasts sharply (asterisks) with the surface of normal COL1. Scale bar, 200 μm


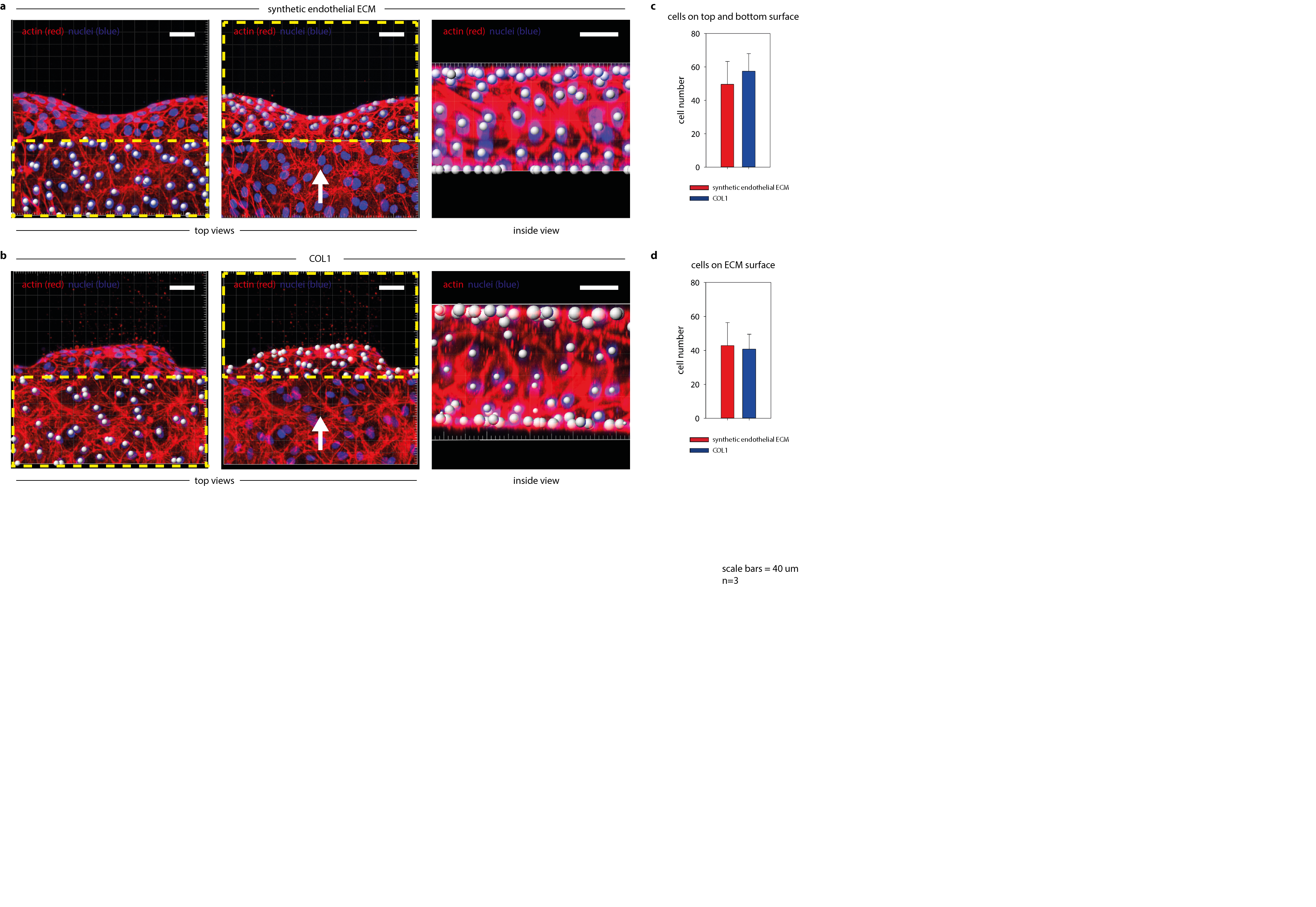


**Supplementary Figure 3|** Quantification of cell numbers on gels (synthetic endothelial ECM and COL1), top and bottom sides. The cell numbers were quantified from confocal microscopy images. **a,** ECs cultured in the synthetic endothelial ECM system. The inside view (right image) was taken in the direction of a white arrow in the middle image and **b,** the COL1 system. The inside view (right image) was taken in the direction of a white arrow in the middle image **c,** Quantified cell numbers on top and bottom surfaces of the microfluidic channels (yellow dotted rectangle in the left image of Supplementary Figures. S3a,b). **d,** Quantified cell numbers on gel surfaces (yellow dotted rectangle in the center image of Supplementary Figures. S3a,b). Scale bars, 40 μm. Error bars represent standard deviation (n = 3).

**
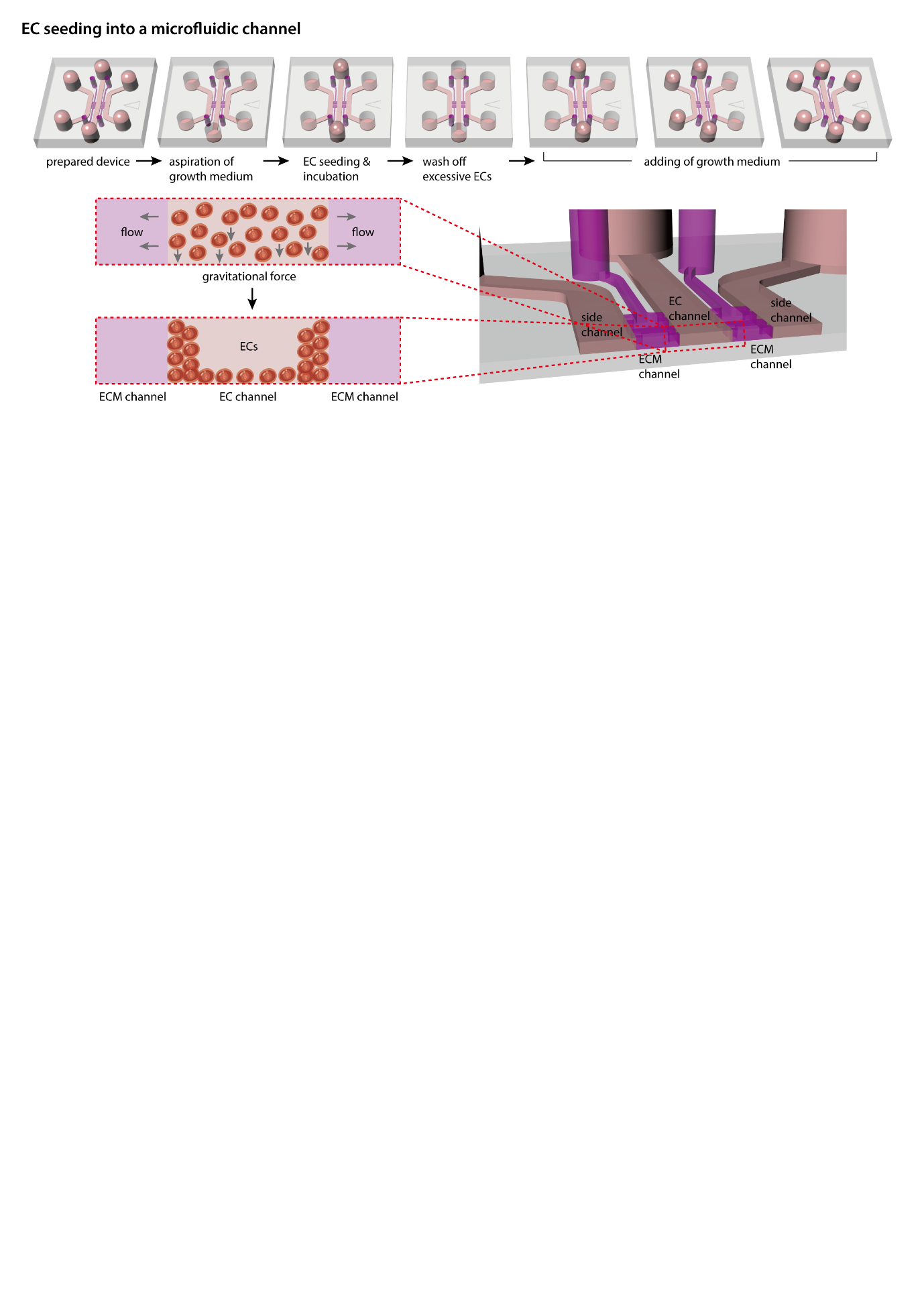
**

**Supplementary Figure 4|** Schematic of seeding procedure of endothelial cells (ECs) into a microfluidic channel. Hydrostatic pressure between the EC channel and both side channels is generated by height difference among the reservoirs of the channels. Due to the pressure difference and the gravitational force, the ECs were initially seeded onto the surface of ECMs and the bottom.

**
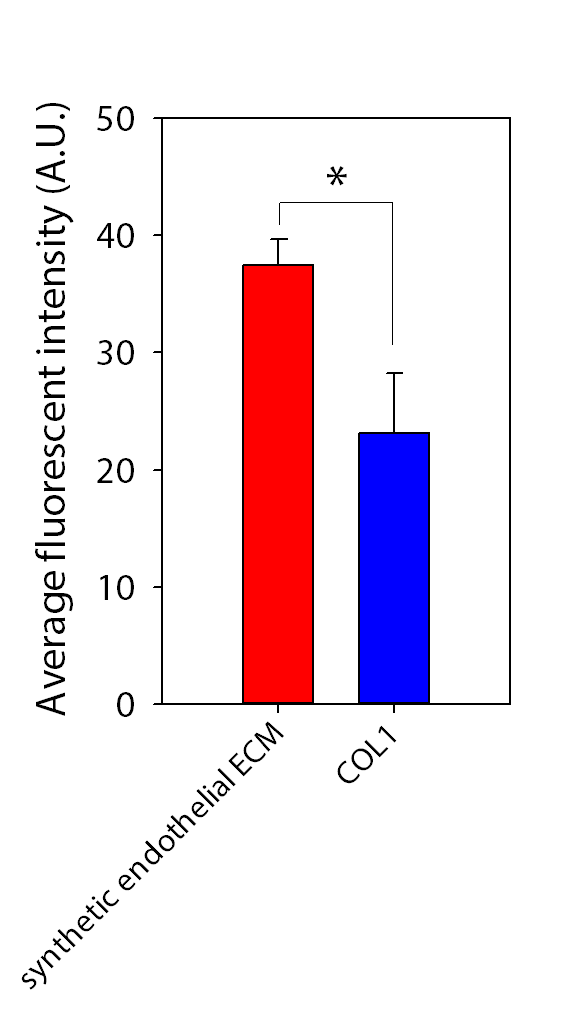
**

**Supplementary Figure 5|** Average fluorescent intensities of the VE-Cadherin expressions in the synthetic endothelial ECM (red bar) and the COL1 (blue bar) systems at culture day5. Error bars represent standard deviation (*P=0.011, n = 3).

**
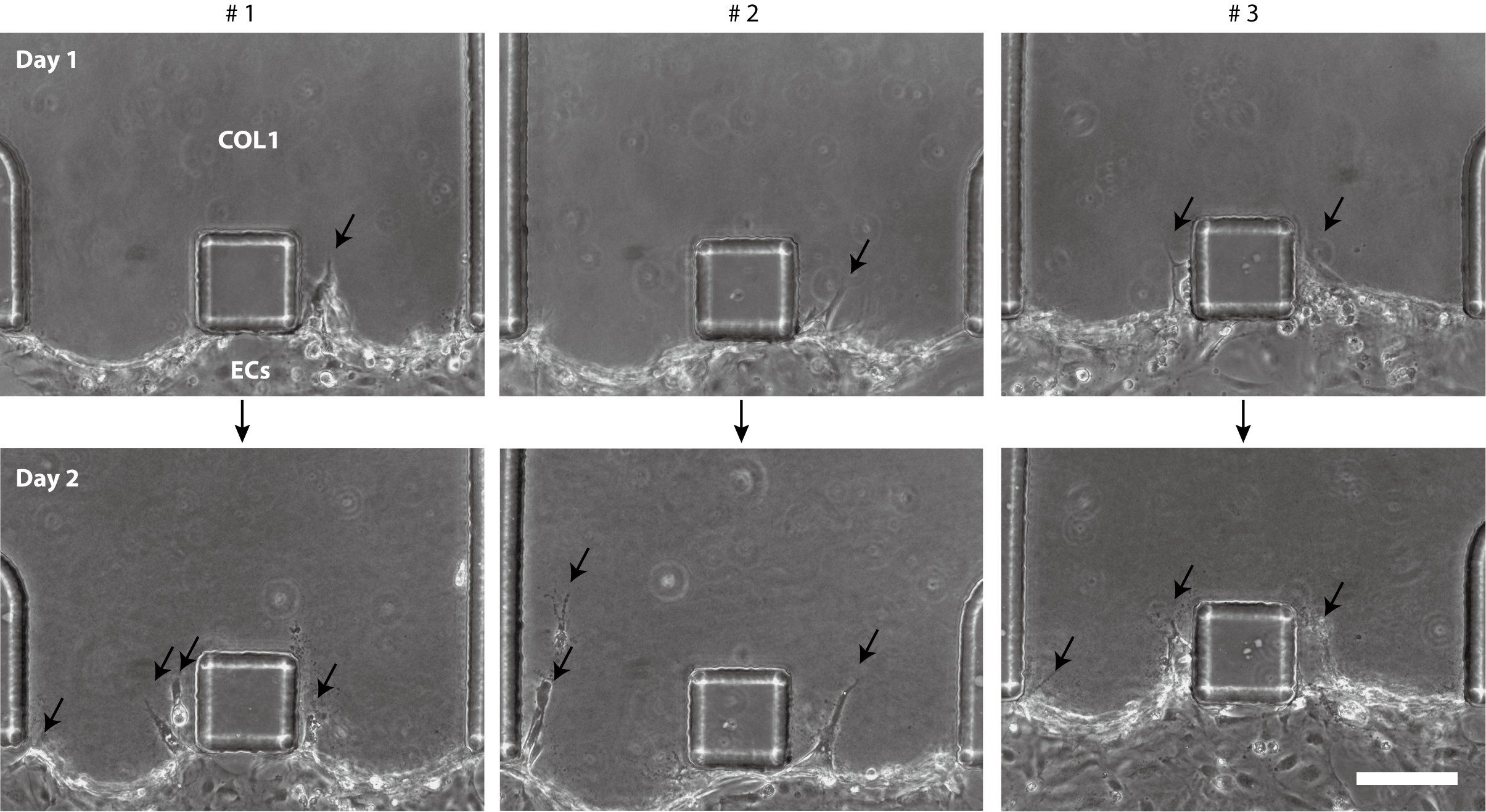
**

**Supplementary Figure 6|** Sprouts were persistingly generated in the COL1 system. Arrows indicate EC sprouting through COL1. Scale bar, 150 μm.

**
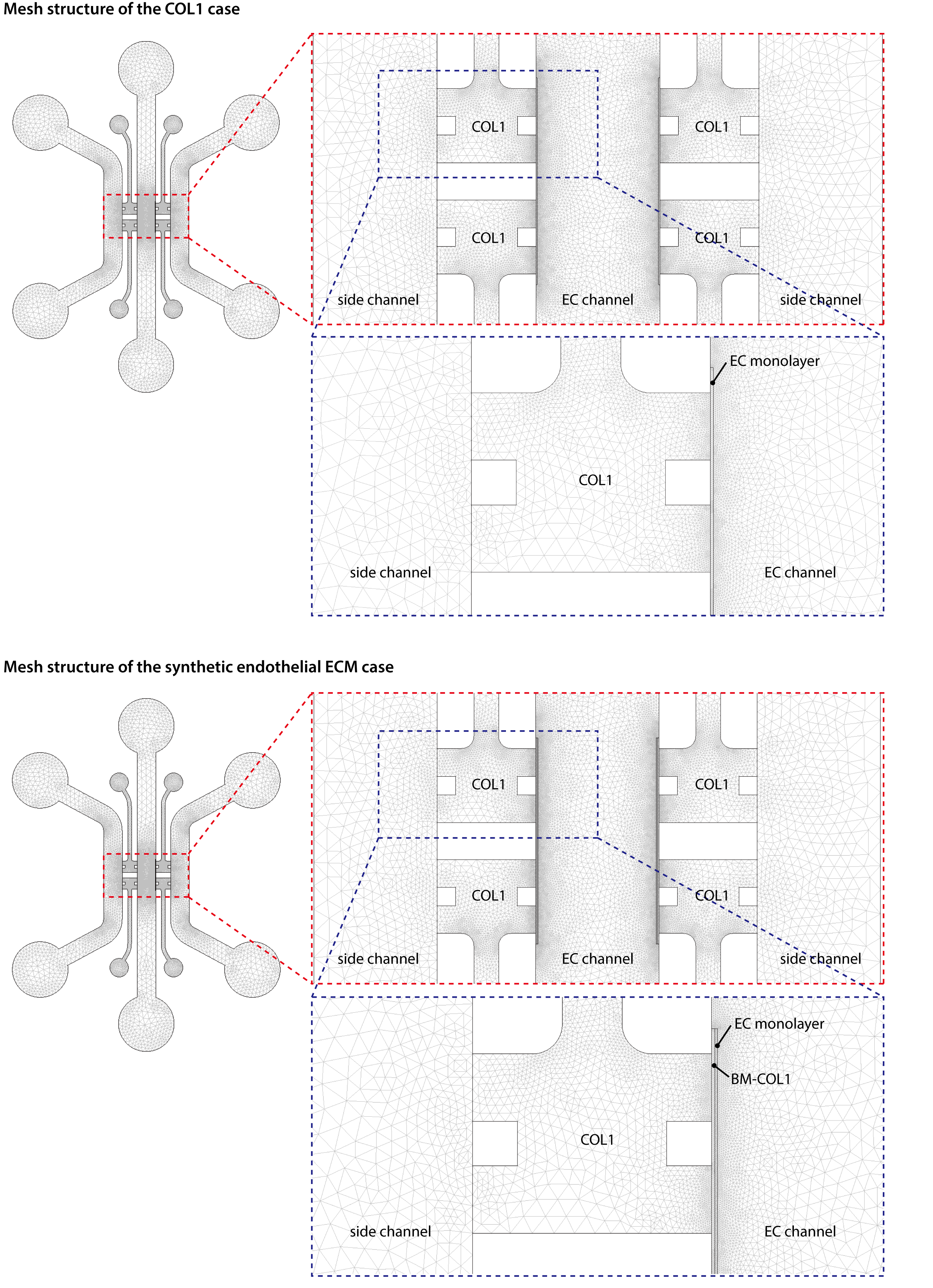
**

**Supplementary Figure 7|** Mesh structures of the simulation in Fig. 3b, and Supplementary Figures 9, 10. These structures were automeshed and refined once. The thicknesses of the EC monolayer and BM-COL1 layer were assumed as 10 μm.

**
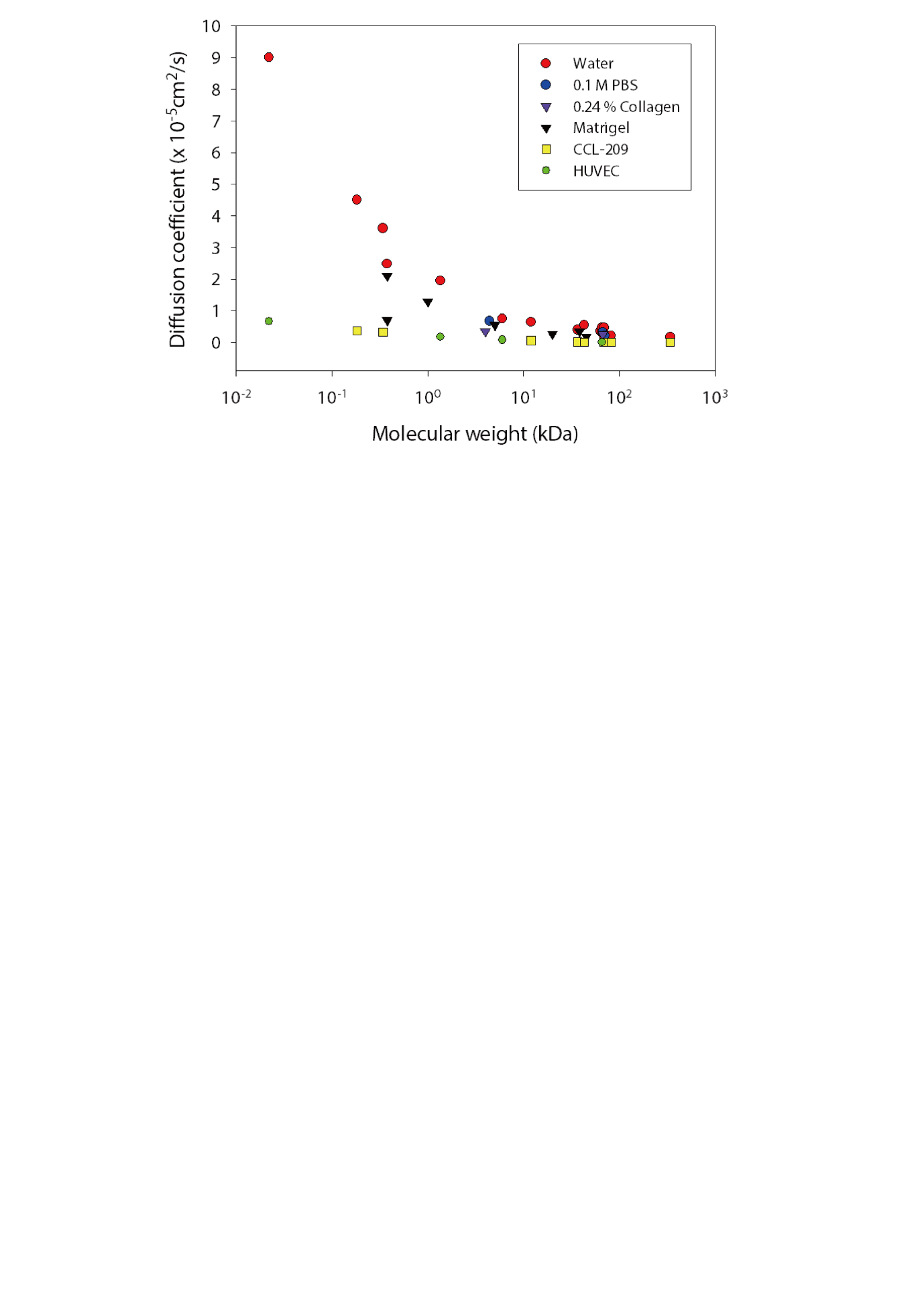
**

**Supplementary Figure 8|** Diffusion coefficients in water (shown in red circles) were obtained from Eaton *et al*. (*Journal of Cellular Physiology*, 1991) and Frisk *et al*. (*9^th^ MicroTAS*, 2005), in 0.1 M PBS (shown in blue circles) were obtained from Pluen *et al*. (*Biophysical Journal*, 1999), in 0.24 % COL1 (shown in purple inverted triangles) were obtained from Ramanujan *et al*. (*Biophysical Journal*, 2002), in Matrigel (shown in black inverted triangles) were obtained from Frisk *et al*. (*9^th^ MicroTAS*, 2005), Kim *et al*. (*Nature Nanotechnology*, 2010), Ciocan *et al*. (*31^st^ IEEE EMBS*, 2009), Chen *et al*. (*Pharmaceutical Research*, 2007), and in EC monolayers (CCL-209 (shown in yellow quadrangles) and HUVEC (shown in green circles)) were obtained from Siflinger-Birnboim *et al*. (*Journal of Cellular Physiology*, 1987) and Eaton *et al*. (*Journal of Cellular Physiology*, 1991), respectively.

**
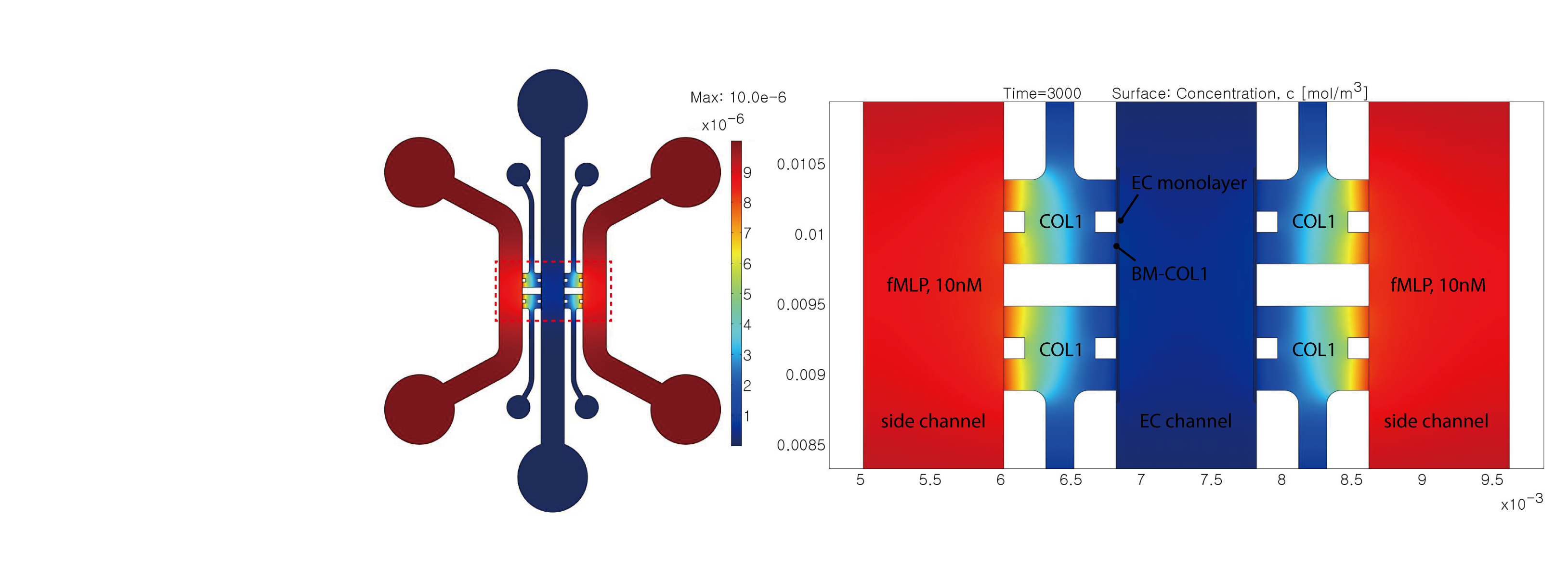
**

**Supplementary Figure 9|** Numerical simulation of the diffusion gradient of N-formyl-methionyl-leucyl-phenylalanine (fMLP, MW: 437.6) in synthetic endothelial ECM system. The concentration profile of the fMLP was captured at 3,000 seconds after the fMLP addition. The right image is magnified from the left image (red dotted rectangle).

**
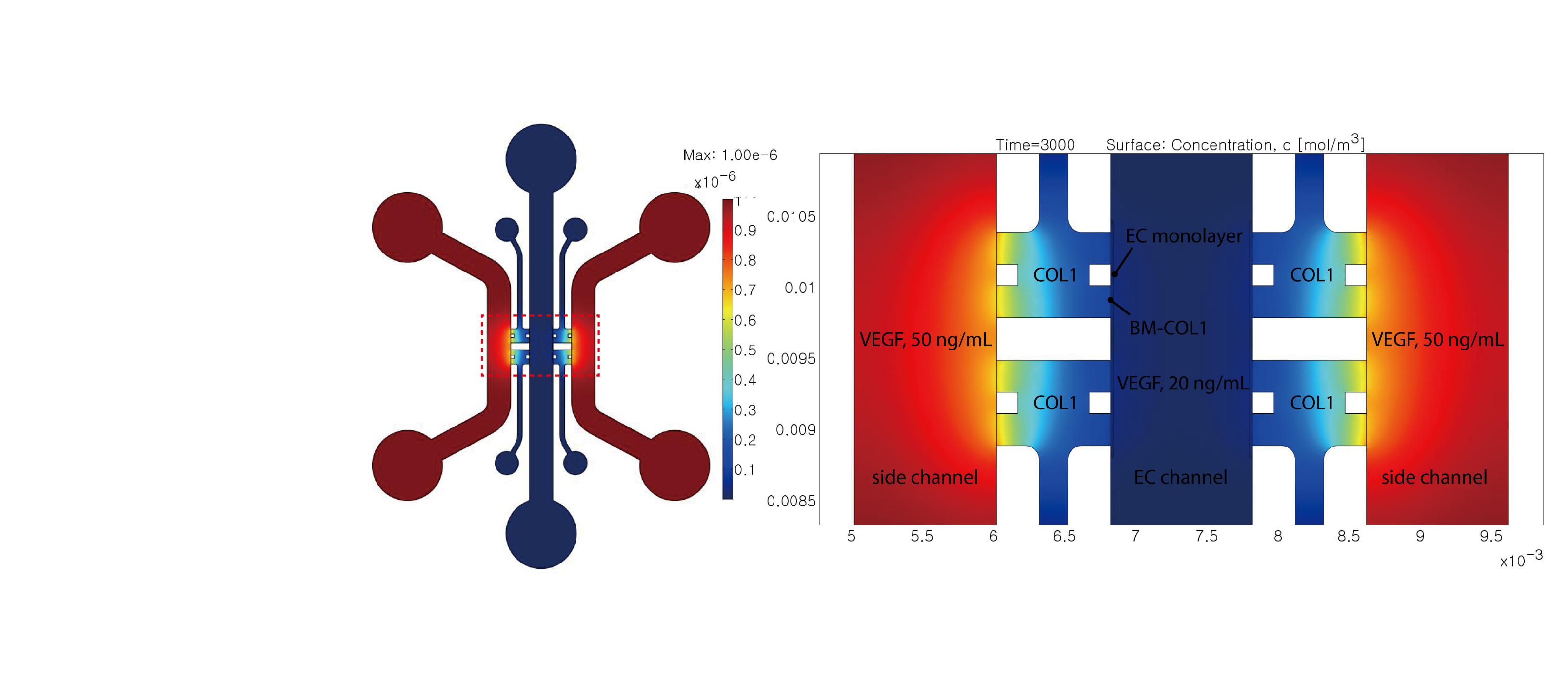
**

**Supplementary Figure 10|** Numerical simulation of the diffusion gradient of vascular endothelial growth factor (VEGF, 40 kDa) in synthetic endothelial ECM system. The concentration profile of the VEGF was captured at 3,000 seconds after the VEGF addition. The right image is magnified from the left image (red dotted rectangle).

**Supplementary Movie 1|** 3D visualization (360° rotational view) of vascular endothelial cadherin (VE-Cadherin) in COL1 system shown in Figure 2c. Green voxel: 3D volumetric image of the VE-Cadherin of ECs; blue voxel: nuclei of the ECs.

**Supplementary Movie 2|** 3D visualization (view along z-axis) of vascular endothelial cadherin (VE-Cadherin) in COL1 system shown in Figure 2c. Green voxel: 3D volumetric image of the VE-Cadherin of ECs; blue voxel: nuclei of the ECs.

**Supplementary Movie 3|** 3D visualization (360° rotational view) of vascular endothelial cadherin (VE-Cadherin) in synthetic endothelial ECM system shown in Figure 2c. Green voxel: 3D volumetric image of the VE-Cadherin of ECs; blue voxel: nuclei of the ECs.

**Supplementary Movie 4|** 3D visualization (view along z-axis) of vascular endothelial cadherin (VE-Cadherin) in synthetic endothelial ECM system shown in Figure 2c. Green voxel: 3D volumetric image of the VE-Cadherin of ECs; blue voxel: nuclei of the ECs.
